# Nutrient sensing and cAMP signaling in yeast: G-protein coupled receptor versus transceptor activation of PKA

**DOI:** 10.15698/mic2021.01.740

**Published:** 2020-10-12

**Authors:** Griet Van Zeebroeck, Liesbeth Demuyser, Zhiqiang Zhang, Ines Cottignie, Johan M. Thevelein

**Affiliations:** 1 Laboratory of Molecular Cell Biology, Institute of Botany and Microbiology, KU Leuven, B-3001 Leuven-Heverlee, Flanders, Belgium.; 2 Center for Microbiology, VIB, Kasteelpark Arenberg 31, B-3001 Leuven-Heverlee, Flanders, Belgium.; † These authors made an equal contribution to this work.

**Keywords:** FRET biosensor, cAMP, PKA, nutrient sensing, GPCR, transceptor, yeast

## Abstract

A major signal transduction pathway regulating cell growth and many associated physiological properties as a function of nutrient availability in the yeast *Saccharomyces cerevisiae* is the protein kinase A (PKA) pathway. Glucose activation of PKA is mediated by G-protein coupled receptor (GPCR) Gpr1, and secondary messenger cAMP. Other nutrients, including nitrogen, phosphate and sulfate, activate PKA in accordingly-starved cells through nutrient transceptors, but apparently without cAMP signaling. We have now used an optimized EPAC-based fluorescence resonance energy transfer (FRET) sensor to precisely monitor *in vivo* cAMP levels after nutrient addition. We show that GPCR-mediated glucose activation of PKA is correlated with a rapid transient increase in the cAMP level *in vivo*, whereas nutrient transceptor-mediated activation by nitrogen, phosphate or sulfate, is not associated with any significant increase in cAMP *in vivo*. We also demonstrate direct physical interaction between the Gap1 amino acid transceptor and the catalytic subunits of PKA, Tpk1, 2 and 3. In addition, we reveal a conserved consensus motif in the nutrient transceptors that is also present in Bcy1, the regulatory subunit of PKA. This suggests that nutrient transceptor activation of PKA may be mediated by direct release of bound PKA catalytic subunits, triggered by the conformational changes occurring during transport of the substrate by the transceptor. Our results support a model in which nutrient transceptors are evolutionary ancestors of GPCRs, employing a more primitive direct signaling mechanism compared to the indirect cAMP second-messenger signaling mechanism used by GPCRs for activation of PKA.

## INTRODUCTION

For all living organisms, but especially for microorganisms*,* nutrients are a major determinant for cell growth and survival. Any change in nutrient availability and composition in the extracellular environment triggers rapid adaptation mechanisms within the cells to properly adjust cellular properties. With respect to the yeast *Saccharomyces cerevisiae*, the presence of fermentable sugar in the extracellular medium has major effects on cell growth and physiology. The cells suppress respiration in favor of ethanol fermentation and show rapid growth, characterized by low levels of reserve carbohydrates, low stress resistance, low expression of stress response element (STRE)-controlled genes, weak cell walls and high expression of ribosomal protein genes. On the other hand, when yeast grows on non-fermentable carbon sources, they proliferate more slowly and show the opposite phenotypes of cells growing on fermentable sugar [1]. Addition of glucose or sucrose to these cells activates Protein Kinase A (PKA) through stimulation of cAMP (cyclic adenosine monophosphate) synthesis, mediated by the G-protein coupled receptor (GPCR), Gpr1, and fructose-1,6-bisphosphate activation of Cdc25/Ras, triggering activation of adenylate cyclase [2, 3]. However, to maintain the high PKA phenotype typical for fermenting cells, a complete fermentable growth medium is required. When fermenting cells are starved for any other essential nutrient, they will arrest in the G1 phase of the cell cycle, enter the stationary phase G0 and acquire a low PKA phenotype, similar to that of cells proliferating slowly on non-fermentable carbon sources [1].

Re-addition of the missing essential nutrient to fermenting stationary-phase cells starved for this nutrient will trigger a similar rapid activation of PKA as addition of a fermentable sugar to respiring cells, and support the re-initiation of rapid fermentable growth. When this upshift is triggered by amino acids, ammonium, phosphate, sulfate, iron or zinc, resupplemented to accordingly-starved fermenting cells, it is not associated with an increase in the cAMP level in spite of the apparent rapid activation of PKA, as inferred from the rapid phosphorylation and adaptation of PKA target systems [4–6]. In this case, no evidence has been found for involvement of nutrient-sensing GPCRs, but rather for high-affinity starvation-induced transporters acting as receptors, or transceptors [7, 8]. This may be consistent with a more primitive activation mechanism of PKA by nutrient transceptors, preceding in evolution the more elaborate mechanisms employed by dedicated receptors and making use of second-messenger signaling [8, 9]. A convenient read-out for rapid activation of PKA is the *NTH1*-encoded neutral trehalase, which is about tenfold activated by direct PKA phosphorylation, and responsible for the rapid mobilization of trehalose during the initiation or stimulation of fermentation [10].

Up to now, all cAMP measurements in the previous studies on nutrient signaling have been performed *in vitro* using cell extracts obtained after rapid filtering, freezing and subzero destruction of enzyme activity with perchloric acid, and using a commercial kit for cAMP determination in the neutralized extracts [11]. Because of the short time courses, the necessity of subzero elimination of cAMP phosphodiesterase activity and the presence of background signals with some commercial cAMP determination kits, these *in vitro* measurements have always remained challenging. More recently, Fluorescence or Förster resonance energy transfer (FRET) based methodologies for real-time *in vivo* monitoring of metabolite levels with excellent temporal resolution have been developed. In 2004, a biosensor was created to monitor levels of cAMP in mammalian cells by tagging the N- and C-terminal ends of the cAMP-binding EPAC (exchange protein activated by cAMP) protein with a FRET donor and acceptor [12, 13]. This system has subsequently been optimized further [14–16]. In 2017, the first functional *S. cerevisiae* variant of the EPAC sensor was developed, based on the original EYFP-EPAC2-ECFP construct of Nikolaev *et al.* [17]. Lately, the research group of Bas Teusink has developed an alternative cAMP sensor for *S. cerevisiae* based on the mammalian sensor of Klarenbeek *et al.* [16], using mTurquoise2del as FRET donor and tdTomato as FRET acceptor [18]. The major advantage of this latest sensor is the fact that the fluorescent proteins used are less pH sensitive, within the physiological intracellular pH-range of the yeast cells, enabling these sensors to report the cAMP concentration more reliably and independent of changes in intracellular pH. Since we are studying nutrient-induced effects known to affect intracellular pH, such as the transient glucose-induced drop in intracellular pH [19–21], this is an important advantage in experiments aimed at measuring *in vivo* changes in the cAMP level.

We have now employed this optimized cAMP sensor for determination of cAMP levels *in vivo* during activation of PKA with different nutrients in yeast. We show that glucose triggers rapid cAMP signaling dependent on the GPCR, Gpr1. On the other hand, nutrient transceptor-dependent activation of PKA with nitrogen (amino acids or ammonium), phosphate or sulfate, in accordingly-starved cells is not associated with any significant increase in cAMP. This is consistent with two different signaling mechanisms for nutrient activation of PKA, either GPCR-mediated cAMP signaling or transceptor-mediated cAMP-independent signaling. Moreover, we show that the Gap1 amino acid transceptor interacts directly with the catalytic subunits (Tpk1-3) of PKA, using an *in vitro* pull-down assay, and that nutrient transceptors contain a conserved consensus sequence, which is also present in the regulatory subunit (Bcy1). These results suggest that transceptors may use a more primitive, direct mechanism for activation of PKA.

## RESULTS AND DISCUSSION

### Glucose activation of PKA is correlated with cAMP signaling

**Figure 1:**
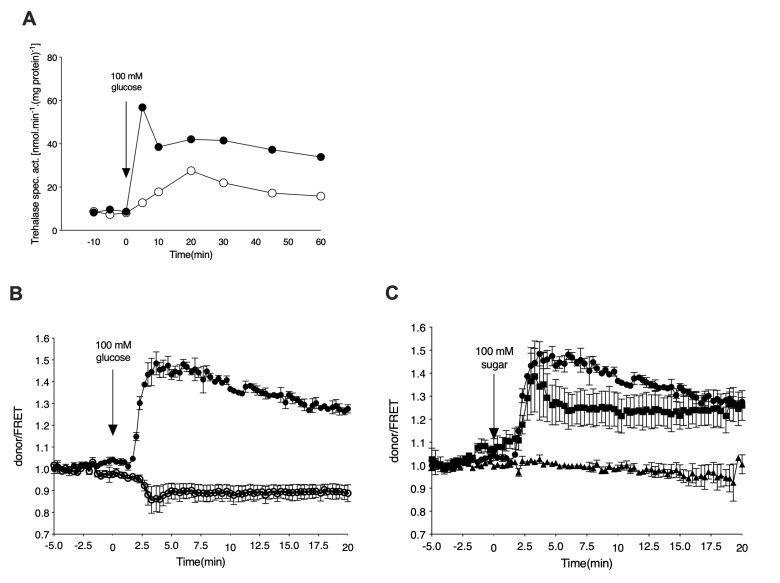
FIGURE 1: Glucose activates trehalase in a Gpr1 receptor and cAMP-dependent manner. **(A)** Activation of the PKA target trehalase after addition of 100 mM glucose to glycerol grown, glucose derepressed cells. Strains: wild type (closed circles) and *gpr1*Δ (open circles). **(B)** Donor/FRET levels for cAMP determination *in vivo* upon addition of glucose to glycerol grown cells. Strains: wild type (closed circles) and *gpr1*Δ (open circles). Mean values of at least three biological replicates, normalised to the value before glucose addition (0 min), are shown with the standard error of the mean. **(C)** Donor/FRET levels for cAMP determination *in vivo* upon addition of 100 mM sugar to glycerol grown wild type cells. Sugars: glucose (circles), sucrose (squares) and fructose (triangles).

In order to monitor cAMP levels upon nutrient addition in a temporally-resolved manner, we have used the EPAC cAMP sensor as optimized by the lab of Bas Teusink [18]. Yeast cells expressing the cAMP sensor were grown in the absence of glucose, using glycerol as the sole carbon source. After 16 to 24 h of growth, the cells were resuspended in fresh medium and either loaded in the microfluidic device or processed further for trehalase activity determination. Addition of 100 mM glucose to glycerol-grown cells caused a rapid increase in trehalase activity also in the cells expressing the cAMP EPAC sensor (**Figure 1A**). This increase was dependent on the GPCR, Gpr1, as shown with the *gpr1*Δ strain (**Figure 1A**). Using the EPAC sensor, we measured a concomitant increase in the cAMP level upon addition of glucose to glycerol-grown cells (**Figure 1B**). Deletion of *GPR1* fully eliminated this cAMP increase (**Figure 1B**). These results are consistent with the previous reports of Gpr1-mediated cAMP signaling being responsible for rapid activation of the PKA target trehalase [22, 23]. Determination of cAMP levels *in vivo* with the EPAC sensor after addition of different sugars showed that glucose and sucrose, but not fructose, triggered a rapid increase in cAMP (**Figure 1C**). This is also consistent with the previously reported sugar specificity of Gpr1 [24].

### Nitrogen, phosphate and sulfate activation of PKA are not correlated with cAMP signaling

**Figure 2:**
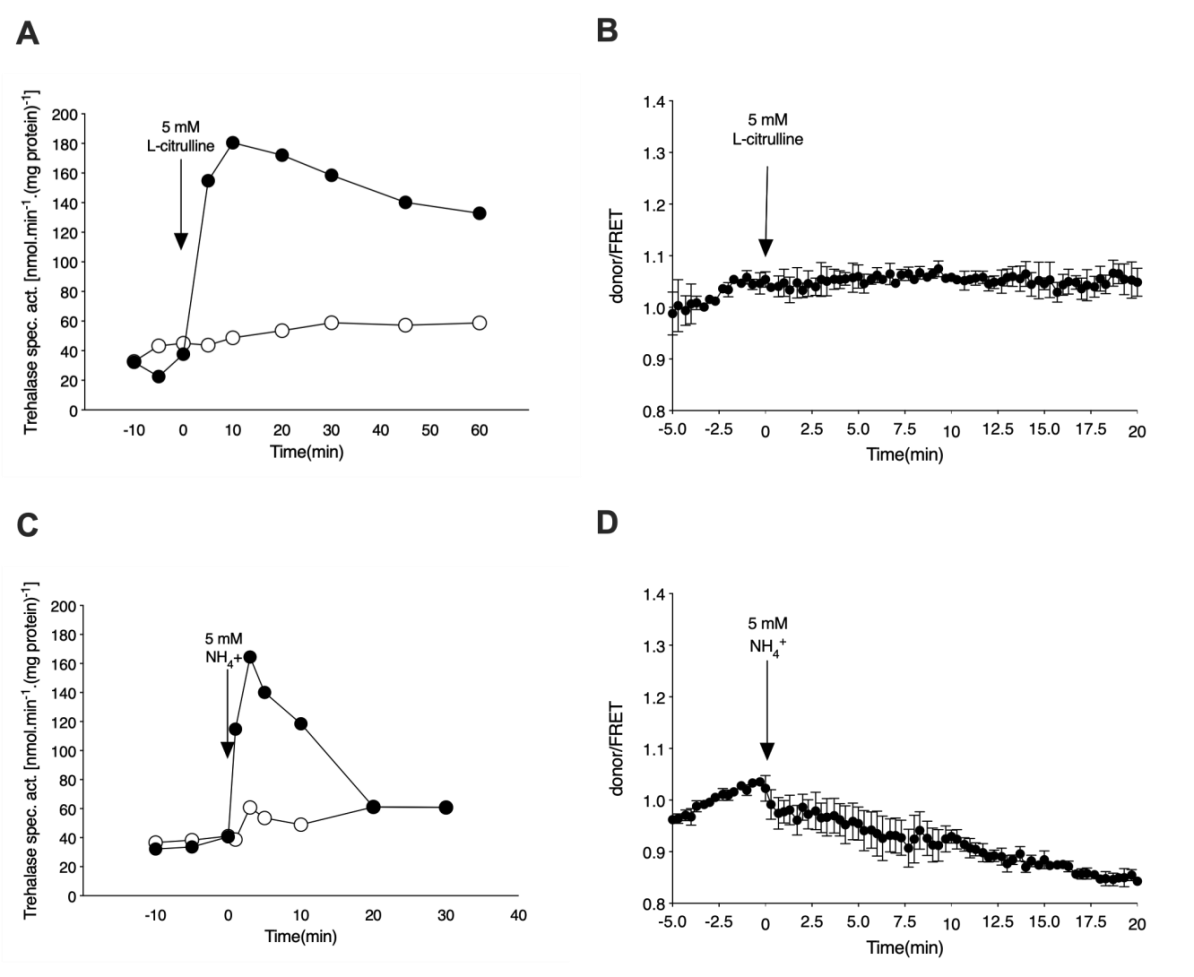
FIGURE 2: Nitrogen activates PKA in a transceptor-dependent, cAMP-independent manner. **(A)** Activation of the PKA target trehalase in nitrogen-starved cells after addition of 5 mM L-citrulline. Strains: wild type (closed circles) and *gap1*Δ (open circles). **(B)** Donor/FRET levels for cAMP determination *in vivo* after addition of 5 mM L-citrulline to nitrogen-starved cells of the wild type strain. **(C)** Activation of the PKA target trehalase in nitrogen-starved cells of the wild type strain after addition of 5 mM (NH_4_)_2_SO_4_. Strains: wild type (closed circles) and *mep2*Δ (open circles). **(D)** Donor/FRET levels for cAMP determination *in vivo* after addition of 5 mM ammonium sulfate to nitrogen-starved cells of the wild type strain.

In the case of nitrogen starvation, yeast cells expressing the cAMP EPAC sensor were incubated for 24h in the absence of any nitrogen source. The nitrogen-starved cells were resuspended in fresh starvation medium and either loaded in the microfluidic device or processed further for determination of trehalase activation by addition of a nitrogen source. Addition of 5 mM of L-citrulline to nitrogen-starved cells triggers a pronounced activation of trehalase also in the cells expressing the cAMP EPAC sensor, and to a similar extent as seen for glucose addition to respiring cells [25] (**Figure 2A**). Moreover, the amino acid transceptor Gap1 is essential for this effect as no increase in trehalase activity is observed in the absence of this protein. As opposed to what is observed for glucose, addition of L-citrulline to nitrogen-starved cells does not trigger any increase in the cAMP level *in vivo*, confirming that the nitrogen source must cause activation of PKA in a cAMP-independent manner (**Figure 2B**). Similarly, addition of ammonium to nitrogen-starved cells causes a rapid increase in trehalase activity also in the cells expressing the cAMP EPAC sensor (**Figure 2C**). Absence of trehalase activation in a *mep2*Δ strain, with Mep2 being the main transceptor for ammonium in *S. cerevisiae*, confirms nutrient specificity [26] (**Figure 2C**). As observed with L-citrulline, the increase in trehalase activity upon re-addition of 5 mM ammonium to nitrogen-starved wild type cells, is not correlated with any increase in the cAMP level (**Figure 2D**). The slight downward trend is possibly due to a technical artefact, such as a focus shift during imaging, rather than reflecting a true change in cAMP levels *in vivo*.

**Figure 3:**
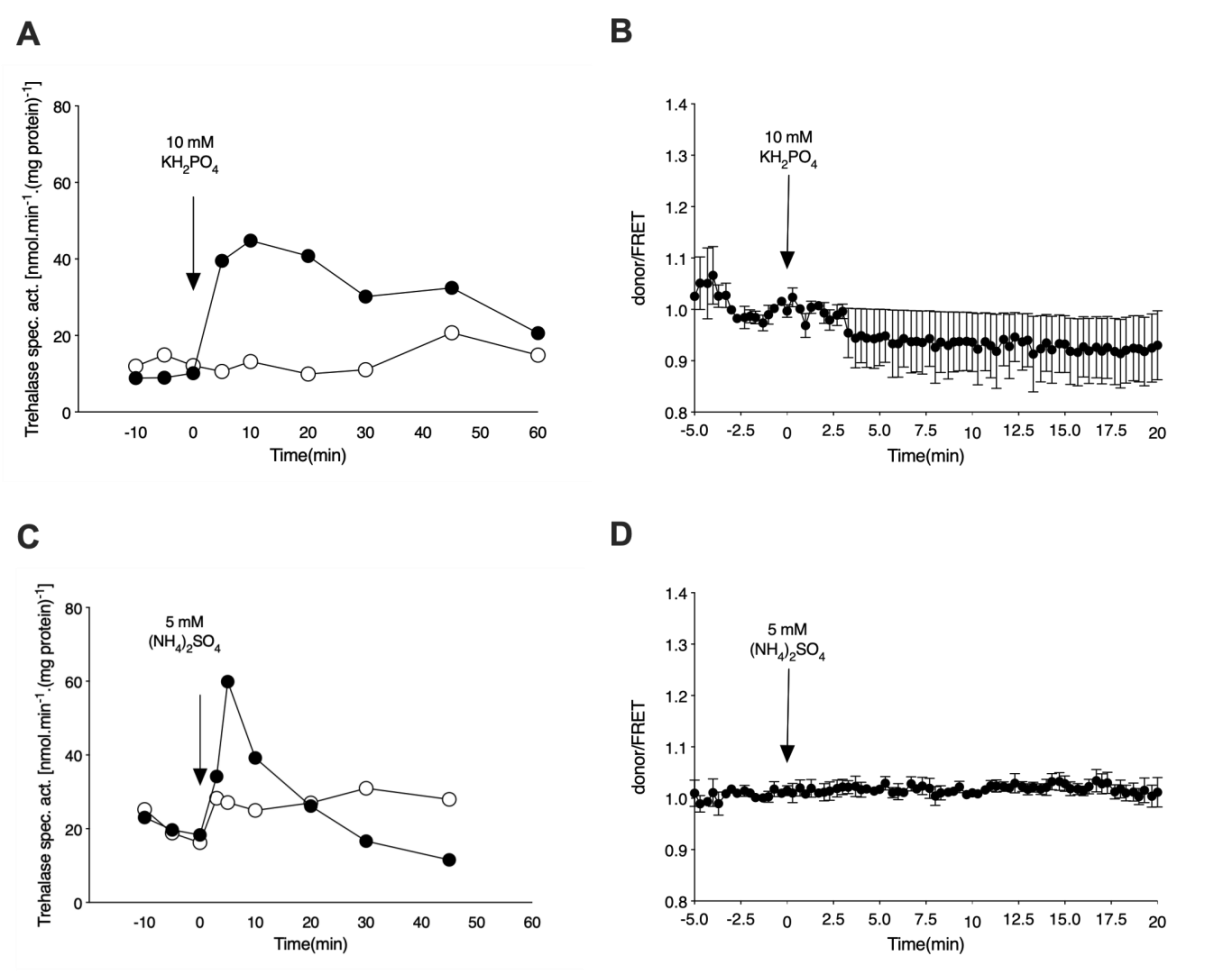
FIGURE 3: Phosphate and sulfate activate PKA in a transceptor-dependent, cAMP-independent manner. **(A)** Activation of the PKA target trehalase in phosphate-starved cells of the wild type strain after addition of 10 mM KH_2_PO_4_. Strains: wild type (closed circles) and *pho84*Δ (open circles). **(B)** Donor/FRET levels for cAMP determination *in vivo* after addition of 10 mM KH_2_PO_4_ to phosphate-starved cells of the wild type strain. **(C)** Activation of the PKA target trehalase in sulfur-starved cells of the wild type strain after addition of 5 mM (NH_4_)_2_SO_4_. Strains: wild type (closed circles) and *sul1*Δ *sul2*Δ (open circles). **(D)** Donor/FRET levels for cAMP determination *in vivo* after addition of 5 mM (NH_4_)_2_SO_4_ to sulfate-starved cells of the wild type strain.

For determination of the cAMP level *in vivo* upon re-addition of phosphate to phosphate-starved cells, the wild type strain expressing the cAMP EPAC sensor was incubated in phosphate starvation medium for 72 h, with daily refreshment of the medium. As in the case of nitrogen-starved cells, the phosphate-starved cells were washed, resuspended in fresh starvation medium and either loaded in the microfluidic device or used for determination of trehalase activation. Addition of 10 mM KH_2_PO_4_ to phosphate-starved cells triggers a rapid increase in the level of trehalase activity also in the cells expressing the cAMP EPAC sensor [27] (**Figure 3A**). The presence of the phosphate transceptor Pho84 is required for this activation as the *pho84*Δ strain does not show any increase in activity upon re-addition of phosphate. Despite the pronounced activation of trehalase, which is known to be mediated by PKA [27, 28], there is no increase in the level of the secondary messenger cAMP, as inferred from the FRET signal, after addition of phosphate to the phosphate-starved cells (**Figure 3B**).

Finally, for determination of the cAMP level *in vivo* upon re-addition of sulfate to sulfate-starved cells, cells expressing the cAMP EPAC sensor were incubated in sulfate starvation medium for 48 h with daily refreshment of the medium. As observed for nitrogen and phosphate addition, the rapid increase in trehalase activity upon re-addition of sulfate (NH_4_)_2_SO_4_) to sulfur-starved cells was not correlated with an increase in the cAMP level *in vivo*, as determined by the FRET sensor (**Figure 3C** and **3D**). As shown previously, absence of the increase in trehalase activity in the absence of the sulfate transceptors Sul1 and Sul2 indicates that these proteins mediate activation of PKA upon sulfate addition to sulfur-starved cells [5].

### Gap1 interacts directly with the catalytic subunits of PKA

**Figure 4:**
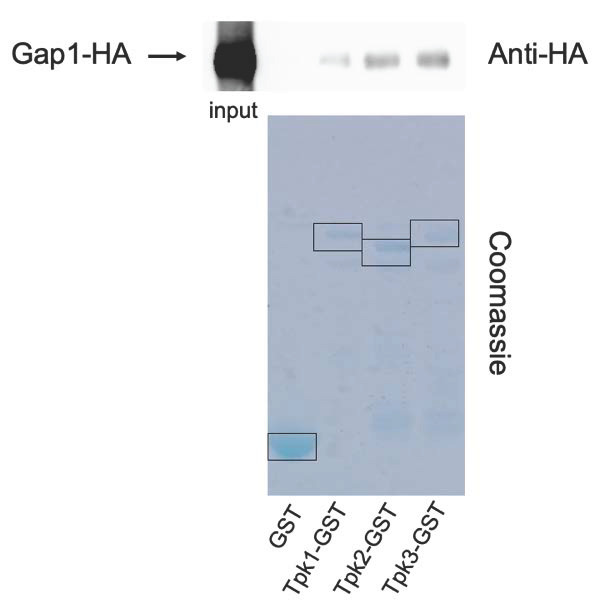
FIGURE 4: Gap1 interacts *in vitro* with the three catalytic subunits of PKA. The indicated GST-tagged proteins were purified from bacteria using glutathione-Sepharose beads and incubated with cell extracts from yeast cells expressing Gap1-HA. GST-pull down assay, followed by SDS-PAGE, Western blotting detection with peroxidase coupled anti-HA antibody demonstrates interaction between Gap1-HA and Tpk1-GST, Tpk2-GST and Tpk3-GST. No Gap1 was recovered using GST alone.

The FRET measurements of *in vivo* cAMP levels during transceptor activation of PKA, suggest that transceptors activate PKA independent of signaling by the classical second messenger of the GPCR-adenylate cyclase pathway. Hence, an alternative activation mechanism of PKA appears to be involved. We explored the possibility that the transceptors would directly interact with PKA to trigger its activation. For that purpose, we have tested *in vitro* interaction of the amino acid transceptor Gap1 with the catalytic subunits of PKA, Tpk1, 2 and 3. HA-tagged Gap1 was expressed in nitrogen-starved yeast cells from a plasmid under its own promotor. The Glutathion-S-transferase (GST) tag as such (as a negative control) and GST-tagged Tpk1, 2 or 3 were expressed in *Escherichia coli*. Gap1-Tpk1, -Tpk2 or -Tpk3 interactions were subsequently tested separately with a GST-pull down assay, in which the GST-tagged catalytic subunits of PKA were precipitated. Co-precipitated HA-tagged Gap1 was detected by Western blot with an anti-HA-HRP antibody. A clear signal of precipitated HA-Gap1 was observed with all three Tpk proteins (**Figure 4**), indicating that Gap1 binds with Tpk1, 2 and 3 *in vitro* (**Figure 4**), while no signal was present for the negative control.

The physical interaction between Gap1 and the catalytic subunits of PKA suggests a simple mechanism for nutrient transceptor activation of PKA. Sequestration of part of the catalytic subunits by the nutrient transceptors, when they are strongly induced during starvation for their substrate, may help to downregulate PKA activity in the nutrient-starved cells. Stationary phase in yeast is generally correlated with a range of physiological properties indicating low PKA activity [1]. Re-addition of the missing nutrient allows its transport by the transceptor and the associated conformational changes in the transceptor may release the catalytic subunits of PKA, resulting in the typical burst in PKA activity, as indicated by the rapid changes in PKA controlled properties, like the activity of trehalase. This may explain why the activation of trehalase in general is closely correlated with the transport activity of the transceptor [8, 25, 29].

### The nutrient transceptors contain a consensus motif also present in Bcy1

**Figure 5:**
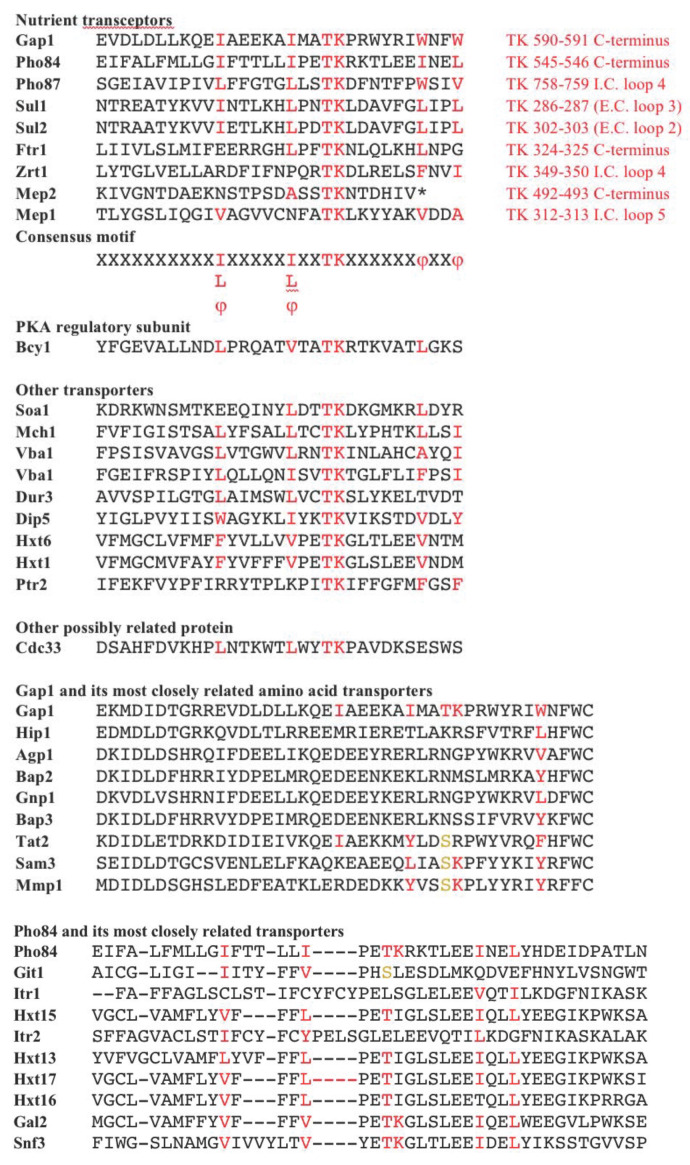
FIGURE 5: Identification of consensus motif in the nutrient transceptors and Bcy1. The consensus sequence (I/L/φ)XXXXX(I/L/φ)XXTKXXXXXXφXXφ is present, at least to a great extent, in all transceptors and in the regulatory subunit of PKA. The conserved motif is also present in a number of other transporters, including the active Hxt transporters of which Hxt1 and Hxt6 are shown as example. The Cdc33 protein, which is required for progression over the nutrient deprivation site in the G1 phase of the cell cycle, like the essential proteins in the cAMP-PKA pathway, also contains the consensus motif. The amino acid transporters most closely related to Gap1 and the transporters most closely related to Pho84 lack the consensus motif, except in the latter case for Gal2 and Snf3.

We have found a common motif in the previously identified transceptors Gap1, Pho84, Pho87, Sul1, Sul2, Ftr1, Zrt1, Mep2, Mep1: (I/L/φ)XXXXX(I/L/φ)XXTKXXXXXXφXXφ (**Figure 5**). The most specific element is the TK motif, which is preceded by a hydrophobic amino acid, preferably leucine or isoleucine, with two random amino acids in between, and this residue is in turn preceded by another hydrophobic amino acid, also preferably leucine or isoleucine, with five amino acid residues in between. The TK motif is followed by two hydrophobic amino acids, with six and two random amino acids in between. The consensus motif is found in the C-terminal tail of the transceptors or in an intracellular loop close to the C-terminus. In the Sul1 and Sul2 transceptors, the consensus motif is predicted to be in an extracellular loop, but the structure prediction for Sul1 and Sul2 is uncertain. This consensus motif is also found in the otherwise totally unrelated protein Bcy1, the regulatory subunit of PKA (**Figure 5**), which is well known to bind the catalytic subunits Tpk1-3. Hence, the presence of this consensus motif may be indicative of physical interaction between a protein and the catalytic subunits of PKA.

We also found the consensus motif in a number of other transporters, including the active Hxt transporters of which Hxt1 and Hxt6 are shown as example (**Figure 5**). This may be indicative for possible interaction with PKA and transceptor functionality. The consensus sequence also appears to be present in Cdc33, which is the translation initiation factor eIF4E and mRNA cap binding protein. The conditional *cdc33* mutant arrests at the restrictive temperature at the nutrient starvation site in the G1 phase of the cell cycle, just like the conditional mutants *cdc25* and *cdc35*, which are deficient in cAMP-dependent PKA activity at the restrictive temperature [30, 31]. The connection between Cdc33 and the cAMP-PKA pathway remained unclear. The presence of the consensus sequence in Cdc33 may indicate that it interacts with the catalytic subunits of PKA and that its inactivation may compromise PKA activity.

The consensus motif is not present in the amino acid transporters most closely related to Gap1 and also not in the transporters most closely related to Pho84 (except for Gal2 and Snf3) (**Figure 5**). This indicates that the consensus motif is not just a common structural or regulatory element in these transporter families. The consensus sequence is also found in the Gal2 galactose/glucose permease and in Snf3, a transporter-like glucose sensor. Connections between the Snf3/Rgt2 glucose sensing pathway and the PKA pathway have been described [32, 33].

The current data seem to support the concept that the consensus motif identified in this work is not just a structural or regulatory element conserved in a family of transport proteins but that it points to a specific regulatory function that may be related to the specific transceptor functionality in sensing and/or signaling the transport of the nutrient by the transceptor. The presence of the consensus motif in the Bcy1 regulatory subunit of PKA (**Figure 5**) and the physical binding of the catalytic subunits to the Gap1 transceptor (**Figure 4**) are consistent with a function of the consensus motif in the interaction with the catalytic subunits Tpk1-3 of PKA. Further research will have to reveal the precise function of this consensus motif.

### Conclusions

We have shown that the optimized EPAC sensor is a convenient tool to measure rapid changes in the cAMP level *in vivo* as a function of time in response to the addition of different nutrients. Using this sensor, we confirm that GPCR-mediated glucose activation of PKA in yeast is correlated with a sharp increase in the level of cAMP as second messenger while transceptor-mediated activation of PKA by other nutrients, including nitrogen, phosphate and sulfate, is not correlated with any increase in the cAMP level *in vivo*. The identification of a consensus motif in the nutrient transceptors that is also present in the Bcy1 regulatory subunit of PKA, and the physical interaction *in vitro* between the Gap1 transceptor and the Tpk1-3 catalytic subunits of PKA, suggest that nutrient transceptor activation of PKA may be mediated by direct release of bound PKA catalytic subunits, triggered by the conformational changes occurring during transport of the substrate by the transceptor. Our results support a model in which nutrient transceptors are evolutionary ancestors of GPCRs, employing a more primitive direct signaling mechanism compared to the indirect cAMP second-messenger signaling mechanism used by GPCRs for activation of PKA.

## MATERIALS AND METHODS

### Strains, plasmids and growth conditions

All strains and plasmids used in this work are summarized in **Table 1** and **2**, respectively. For FRET measurements, the wild type strain was transformed with the mTurq2del-EPACdDEPCD-tdTomato plasmid (*URA3*) kindly provided by Bas Teusink (VU Amsterdam) [18]. For the GST-pull down experiments, the *gap1*Δ strain was transformed with a plasmid expressing HA-tagged Gap1. For all experiments, cells were cultured under shaking at 200 rpm and at 30°C till exponential phase (OD_600_ 1.5-2). Glucose-derepressed cells were grown in YPGly medium containing 2% bacteriological peptone, 1% yeast extract and 2% glycerol. Nutrient-starved fermenting cells were pregrown in minimal medium, containing 0.17% (w/v) Difco yeast nitrogen base without amino acids and without ammonium sulfate, 0.5% (w/v) ammonium sulfate and 2% (w/v) glucose, containing a complete supplement mixture without uracil (SD-Ura) until exponential phase (OD_600_ 1.5-2). They were harvested and resuspended in the appropriate starvation medium containing 4% glucose, and further incubated with shaking at 30°C. Nitrogen-starved cells were obtained by incubation for 24 h in nitrogen starvation medium containing 0.17% (w/v) Difco yeast nitrogen base without amino acids and without ammonium sulfate. Phosphate-starved cells were obtained by incubation for 72 h (with medium refreshment every 24 h) in phosphate starvation medium (0.57% yeast nitrogen base without phosphate). Sulfur-starved cells were obtained by incubation for 48 h (with medium refreshment every 24 h) in sulfur starvation medium as previously described [5].

**TABLE 1.** *S. cerevisiae* strains used in this work.

**Strain name**	**Genotype**	**Source**
BY4742	MATα *his3 leu2 lys2 ura3*	[34]
HS14	BY MATα *gpr1::HIS3 leu2 lys2 ura3*	MCB collection
HK14	BY MATα *sul1::KanMx sul2::KanMX his3 leu2 lys2 ura3*	[5]
Σ*1278b*	MATα *ura3*	[35]
JT20867	Σ1278b MATα *gap1::KanMX ura3*	[25]
JT21033	Σ1278b MATα *mep2::KanMX ura3*	[26]
MB191	MATa *pho3-1 ade2 leu2 his3 trp1 ura3*	[36]
MB192	MATa *pho3-1 ade2 leu2 trp1 ura3 pho84::HIS3*	[36]

**TABLE 2.** Plasmids used in this work.

**Plasmid name**	**Insert**	**Source**
pFL38-Gap1-HA	Gap1-HA	2617 MCB collection
pGEX-GST	GST	993 MCB collection
pGEX-Tpk1-GST	Tpk1-GST	1301 MCB collection
pGEX-Tpk2-GST	Tpk2-GST	1160 MCB collection
pGEX-Tpk3-GST	Tpk3-GST	1161 MCB collection

### Trehalase activity

Trehalase activity as a function of time in response to nutrient addition was determined in cell extracts as previously described [25]. The specific activity of trehalase is expressed as nmol glucose liberated. min^-1^. (mg protein)^-1^. Total amount of protein in the samples was determined using the standard Lowry method. Time-course experiments of nutrient-induced trehalase activation were repeated at least three times, representative results are shown for these comparisons between collections of independent data points.

### Ratiometric FRET measurements

#### Microfluidics and imaging

The CellASIC ONIX2 Microfluidic Platform from Millipore for automated addition of compounds to cells during continuous imaging by microscopy was used, as previously described [37]. The cells were loaded into a chamber of a microwell plate (Y04C, Merck), and liquid was continuously fluxed through the cell chamber. The first well was filled with the appropriate nutrient starvation medium, the second well with starvation medium supplemented with the missing nutrient. The first liquid was fluxed for 10 min, after which we switched to the second liquid for another 20 min. During fluxing, images of the cells in the donor and FRET channels were taken every 20 s. The donor fluorophore was visualized using the 458 nm laser line, excitation DM458/515, SDM560 and emission BA480-495 filter. FRET was monitored using the 458 nm laser line and emission BA575-675 filter. Images were taken by scanning at 8.0 µs/pixel and the 60x UPlanSApo (NA 1.35) objective lens was used in combination with a digital zoom of 1.5x.

#### Data analysis

The FRET_R_ ratios were calculated for an average of 20 cells by dividing the corrected acceptor value by the corrected donor value, as previously described [37]. Correction was achieved by subtracting the background and the autofluorescence signal intensity from the sample signal intensity of every cell. In order to visually compare the response of all cells, the values were divided by the average of the corrected values taken a short time period before addition of the compound. For the EPAC sensor, the reverse ratios are shown, since an increase in cAMP results in a decrease in FRET. Mean values of at least three biological replicates, normalised to the value before sugar addition (0 min), are shown with the standard error of the mean.

### GST-pull down

#### Expression and purification of GST-tagged proteins from E. coli

Proteins were expressed in the *E*. *coli* strain BL21 and expression induced by addition of 0.3 mM IPTG (final concentration) and cells were harvested and washed once with ice-cold PBS buffer. Cells were then resuspended in 5 ml of lysis buffer (PBS 1x, 0.4% Triton X-100, 2 mM MgCl_2_, 1 mM EDTA pH 8.0, 2 mM DTT, 0.2 mg/ml lysozyme and protease inhibitor mix, complete EDTA free, Roche) and incubated on ice for 15 min. Lysis was completed by 3x15 s pulses of sonication. Lysates were clarified by centrifugation for 10 min at 12,000 *g* at 4°C. The resulting supernatant fraction was incubated with 400 µl of a 50:50 slurry of glutathione sepharose beads (GEHealthcare) (pre-equilibrated in wash buffer (PBS 1x, 0.1% Triton X-100, 2 mM MgCl_2_, 1 mM EDTA, 1 mM DTT)) in a rollerdrum for 1 h at 4°C. Beads were collected by centrifugation at 500 *g* for 2 min at 4°C and washed 5 times with wash buffer.

#### Expression and purification of HA-tagged proteins from yeast

Cells were harvested and washed once with ice-cold PBS buffer. Cells were resuspended in 500 µl ice-cold lysis buffer (PBS 1x, 0.1% Triton X-100, 10% glycerol, 2.5 mM MgCl_2_, 1 mM EDTA, 1 mM DTT, 10 mM NaF, 0.4 mM Na_3_VO_4_, 0.1 mM b-glycerophosphate, containing protease inhibitor mix, complete EDTA free, Roche). Glass beads were added and cells were lysed by vigorous vortexing (4x1min, with cooling on ice in between). Lysates were clarified by centrifugation at 12,000 *g* for 10 min at 4°C. Supernatant was transferred to a new microcentrifuge tube and centrifuged for a second time at 12,000 *g*. Clarified extracts were kept on ice for further use in pull down assays.

#### GST-pull down assay

GST fusion proteins were extracted from BL21 *E. coli* cells as described. Beads were resuspended in 500 µl binding buffer (PBS 1x, 0.05% Triton X-100, 0.1 mM DTT). Yeast cell extracts were prepared as described and clarified extracts were incubated for 60 min at 4°C with 50 µl glutathione sepharose beads (GE Healthcare) to reduce aspecific binding. Beads were collected with a brief spin at 500 *g* and the resulting supernatant was incubated with equal amounts of beadbound purified GST fusion proteins, prepared as described. After a 2 h incubation at 4°C, samples were allowed to stand for 5 min on ice. The sedimented beads were washed three times with PBS-T (PBS 1x, 0.1% Triton X-100). Finally, proteins were solubilized by adding SDS sample buffer, separated by SDS-PAGE, and visualized by Coomassie staining or immunoblotting with HRP-coupled anti-HA antibody.

## Abbreviations:

cAMP: – cyclic adenosine monophosphate,
EPAC: – exchange protein activated by cAMP,
FRET: – fluorescence or förster resonance energy transfer,
GPCR: – G-protein coupled receptor,
GST: – Glutathion-S-Transferase,
PKA: – protein kinase A.
